# Studying the Structural Significance of Galectin Design by Playing a Modular Puzzle: Homodimer Generation from Human Tandem-Repeat-Type (Heterodimeric) Galectin-8 by Domain Shuffling

**DOI:** 10.3390/molecules22091572

**Published:** 2017-09-19

**Authors:** Anna-Kristin Ludwig, Malwina Michalak, Nadya Shilova, Sabine André, Herbert Kaltner, Nicolai V. Bovin, Jürgen Kopitz, Hans-Joachim Gabius

**Affiliations:** 1 Institut für Physiologische Chemie, Tierärztliche Fakultät, Ludwig-Maximilians-Universität, Veterinärstraße 13, 80539 München, Germany; an.ludwig@lmu.de (A.-K.L.); sabine.andre@lmu.de (S.A.); kaltner@lmu.de (H.K.); 2 Abteilung für Angewandte Tumorbiologie, Pathologisches Institut, Klinikum der Ruprecht-Karls-Universität, Im Neuenheimer Feld 224, 69120 Heidelberg, Germany; Malwina.Michalak@med.uni-heidelberg.de; 3 Shemyakin & Ovchinnikov Institute of Bioorganic Chemistry, Russian Academy of Sciences, ul Miklukho-Maklaya 16/10, Moscow 117997, Russia; pumatnv@gmail.com

**Keywords:** adhesion, glycoprotein, haemagglutination, lectin, sialylation

## Abstract

Tissue lectins are emerging (patho)physiological effectors with broad significance. The capacity of adhesion/growth-regulatory galectins to form functional complexes with distinct cellular glycoconjugates is based on molecular selection of matching partners. Engineering of variants by changing the topological display of carbohydrate recognition domains (CRDs) provides tools to understand the inherent specificity of the functional pairing. We here illustrate its practical implementation in the case of human tandem-repeat-type galectin-8 (Gal-8). It is termed Gal-8 (NC) due to presence of two different CRDs at the N- and C-terminal positions. Gal-8N exhibits exceptionally high affinity for 3′-sialylated/sulfated β-galactosides. This protein is turned into a new homodimer, i.e., Gal-8 (NN), by engineering. The product maintained activity for lactose-inhibitable binding of glycans and glycoproteins. Preferential association with 3′-sialylated/sulfated (and 6-sulfated) β-galactosides was seen by glycan-array analysis when compared to the wild-type protein, which also strongly bound to ABH-type epitopes. Agglutination of erythrocytes documented functional bivalency. This result substantiates the potential for comparative functional studies between the variant and natural Gal-8 (NC)/Gal-8N.

## 1. Introduction

Glycosylation of lipids and proteins is a complex, non-random event that establishes a molecular fingerprint on cells and in tissues [1,2,3,4,5,6,7]. The more we learn about glycan structures and their changes upon differentiation or disease processes through technical advances [8,9,10,11], the higher becomes the likelihood of the validity of the concept of a biological meaning of glycan determinants (sugar code; [12]). The implied potential of glycans as signals can be realized by recognition processes. Fittingly, the application of sugar receptors (lectins) from plants and invertebrates as tools for glycan detection attests the operativeness of glycan–protein interactions [13,14,15,16,17,18,19,20].

The broad success of this application underscores the manifold opportunities residing in functional pairing between glycans and endogenous lectins in (patho)physiology, and, indeed, human lectins are emerging as potent effectors [21,22,23]. Apparently, as is the case for other types of receptors, the interaction brings matching lectin and glycoconjugate together. Its specificity stems from features on the levels of the glycan, the type of scaffold (protein or sphingolipid), and the lectin, so that pairs of distinct cellular glycoconjugates and their lectins find each other to form functional complexes. In addition to their direct contact involving molecular complementarity between sugars and peptide regions [24], topological factors on both sides are assumed to underlie the exquisite selectivity of mutual target selection in order to translate glycan-encoded information into cellular activities [25]. They encompass presentation of lectin-binding epitopes in clusters by branching or by spatial vicinity in microdomains as well as of corresponding display of carbohydrate recognition domains (CRDs). Of note, a lectin’s modular architecture thus appears as crucial for bringing the binding partners together. Although this sounds perfectly reasonable, the given hypothesis requires solid experimental backing.

Looking at the sugar, the preparation of multivalent glycosides by chemical synthesis has made valuable tools available [26,27,28,29,30], and these efforts teach a salient lesson on what to do on the protein side. The rational engineering of variants of tissue lectins is the equivalent route toward the given end. With focus on topological aspects, a change in lectin design, i.e., its mode of CRD presentation, can become as powerful a means to delineate structure-function relationships as glycoclusters already are. Our report exemplifies this strategy by turning a human tandem-repeat-type lectin with two different CRDs into a covalently linked homodimer. If the ensuing work with the variant reveals an interesting glycan specificity, then respective applications in glycoconjugate monitoring can come as added value.

**Figure 1:**
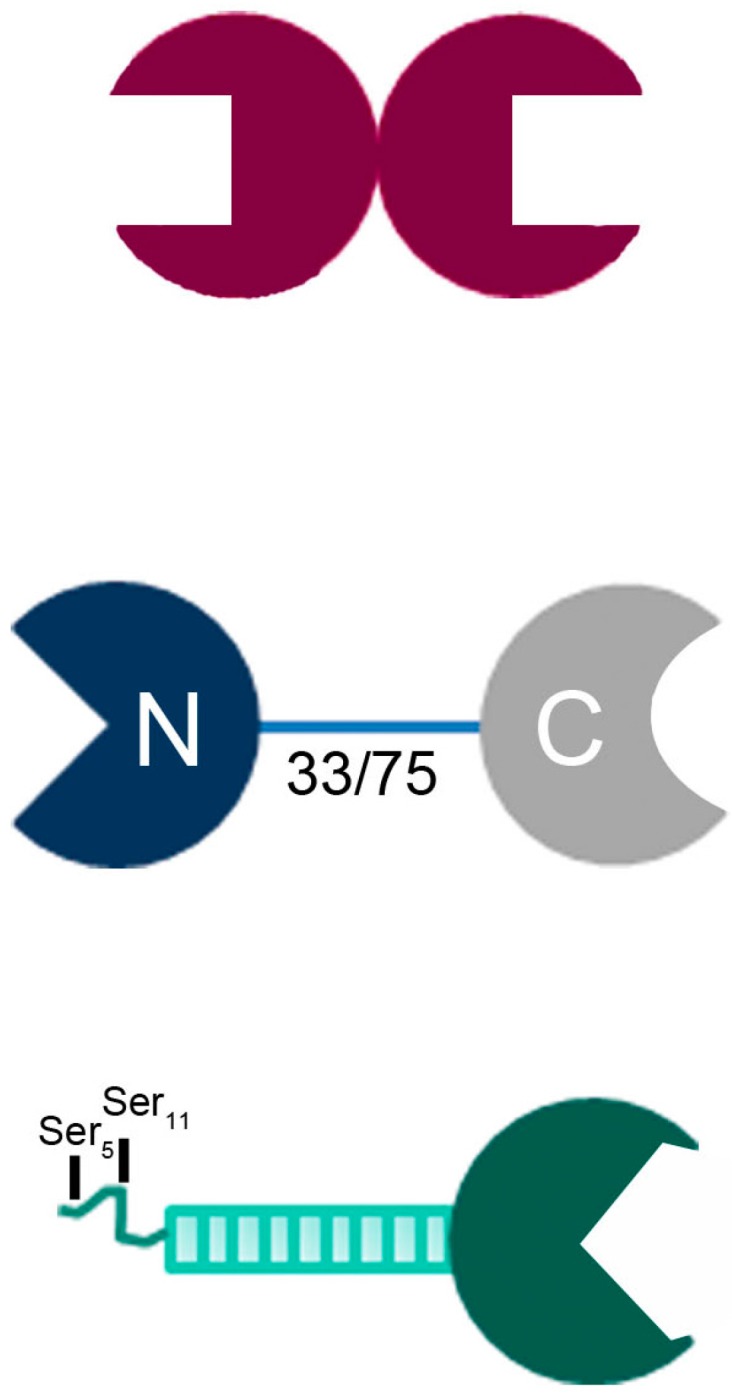
Illustration of the structural design of the three classes of vertebrate galectins, i.e., non-covalently associated homodimer (proto-type), covalently linked heterodimer (with natural variability of linker length by alternative splicing, here given in number of amino acids, for human Gal-8; tandem-repeat-type) and trimodular combination of a C-terminal CRD with nine non-triple helical collagenous repeats and an N-terminal peptide with two sites for serine phosphorylation (chimera-type).

We here focus on a member of the family of adhesion/growth-regulatory galectins, which share the fold (β-sandwich), the ligand class (β-galactosides), and a sequence signature for ligand contact. Three classes of their structural organization are commonly found in vertebrates (Figure 1) [31,32,33]. In contrast to non-covalently associated homodimers shown in the top part of Figure 1, two different CRDs are combined in one protein in tandem-repeat-type family members (middle part). Galectin-8 (Gal-8) is such a protein [34]. Physiologically, it is a matricellular protein with a broad range of activities on immune, endothelial, and bone cells, and is also present in tumor cells (for recent examples, please see [35,36,37,38]). The sequence differences between its two CRDs (Gal-8N, Gal-8C, forming the Gal-8 (NC) wild-type protein) place them rather far apart in the phylogenetic tree [32,39]. As further consequence, these deviations translate into a preference for sialylated/sulfated β-galactosides for Gal-8N, reaching an affinity in the nM range [40,41,42,43,44,45,46,47,48]. Obviously, the engineering of a homodimeric (proto-type-like) Gal-8 variant with two Gal-8N units, termed Gal-8 (NN) in contrast to Gal-8 (NC) used for the wild-type protein, would generate a tool that can enable the determination of whether and then which lectin properties are affected by turning the NC into an NN form. An illustrative example is the comparative characterization of counterreceptors, hereby addressing the fundamental issue of the significance of combining two different CRDs in contrast to the proto-type design of homodimers (Figure 1). This work on human Gal-8 extends previous efforts on the murine homologue used as platelet activator and T cell mitogen [36,49].

In this report, we first describe cDNA tailoring and production of the human homodimeric Gal-8 (NN) variant protein, followed by presenting results of the analysis of its biochemical characteristics, its binding profile in a glycan array, as well as activity in aggregation and tumor cell proliferation assays. This study thus serves two purposes: (i) to turn human tandem-repeat-type Gal-8 (with two different CRDs) into a homodimeric display, and, more generally; (ii) to illustrate the feasibility and perspectives of generation of new tools by structural re-design of an endogenous lectin.

## 2. Results

### 2.1. Protein Production and Characterization

#### 2.1.1. Engineering and Yield

cDNA amplifications were directed to engineer sequences that encode Gal-8 (NN) variants with two N-type CRDs separated by the standard (short: S, 33 aa) or long (L, 75 aa) linkers or connected directly. Screening of production of the human proteins directed from these cDNAs was done in extracts of transfected bacteria by gel electrophoresis and Western blotting. Soluble protein was found exclusively in the case of Gal-8 (NN) (without linker). Its purification under conditions found to be optimal (pET-24a, 22 °C, 100 μM isopropyl-β-d-thiogalactopyranoside (IPTG)) yielded about 3 mg/L. Compared to quantities of about 17–20 mg/L for wild-type Gal-8S/L (NC) proteins and 25 mg/L for Gal-8N, this yield is rather small. As done for these proteins, purification could be based on affinity chromatography with resin-presented lactose so that the canonical binding activity was obviously maintained in the variant.

#### 2.1.2. Structural Characterization

**Figure 2:**
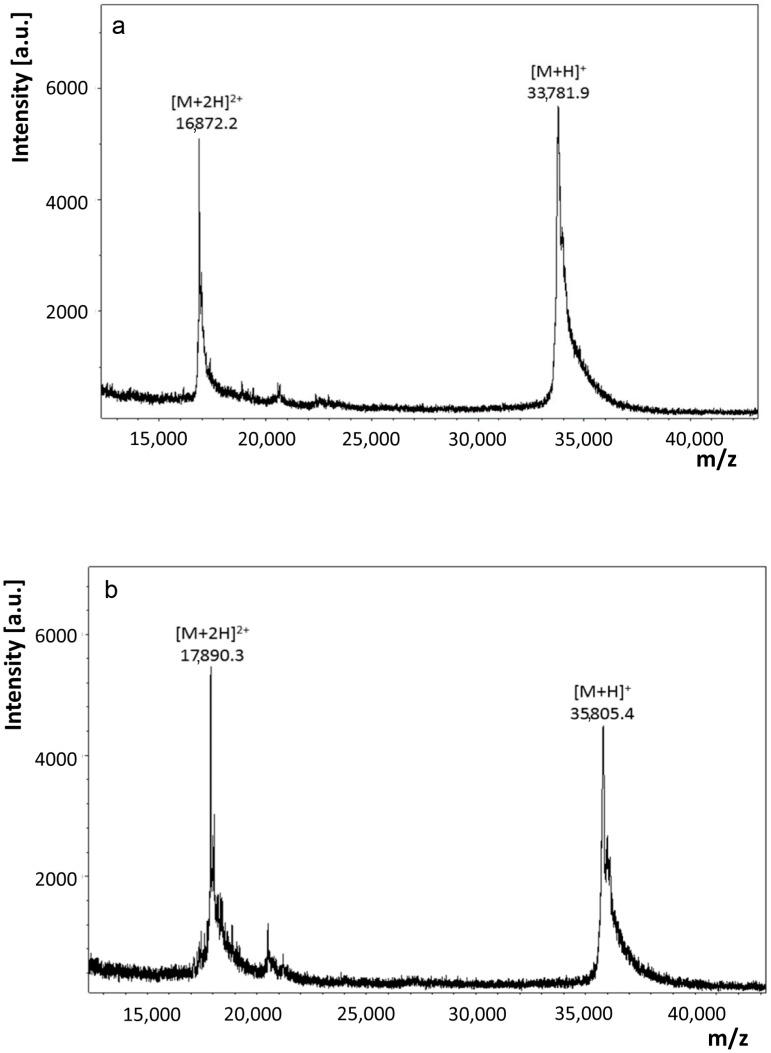
Mass determination of Gal-8 (NN) (**a**) and wild-type Gal-8S (NC) (**b**) by matrix assisted laser desorption ionization-time of flight mass spectrometry (MALDI-TOF MS). The two peaks represent the singly and doubly charged molecular ions of the studied protein.

**Figure 3:**
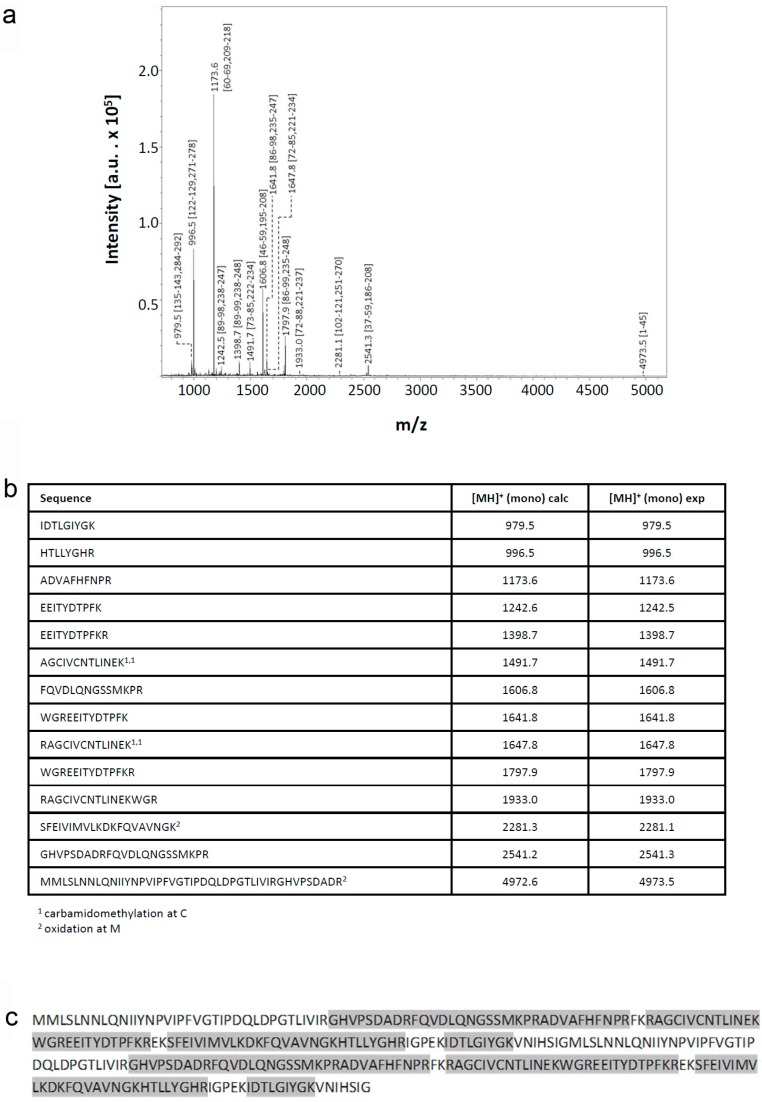
Mass spectrometric fingerprinting of peptides obtained by treatment of Gal-8 (NN) with trypsin. (**a**) The annotated spectrum; (**b**) the list of detected peptides with their calculated (calc) and experimentally (exp) measured mass values and (**c**) the sequence coverage are shown.

**Figure 4:**
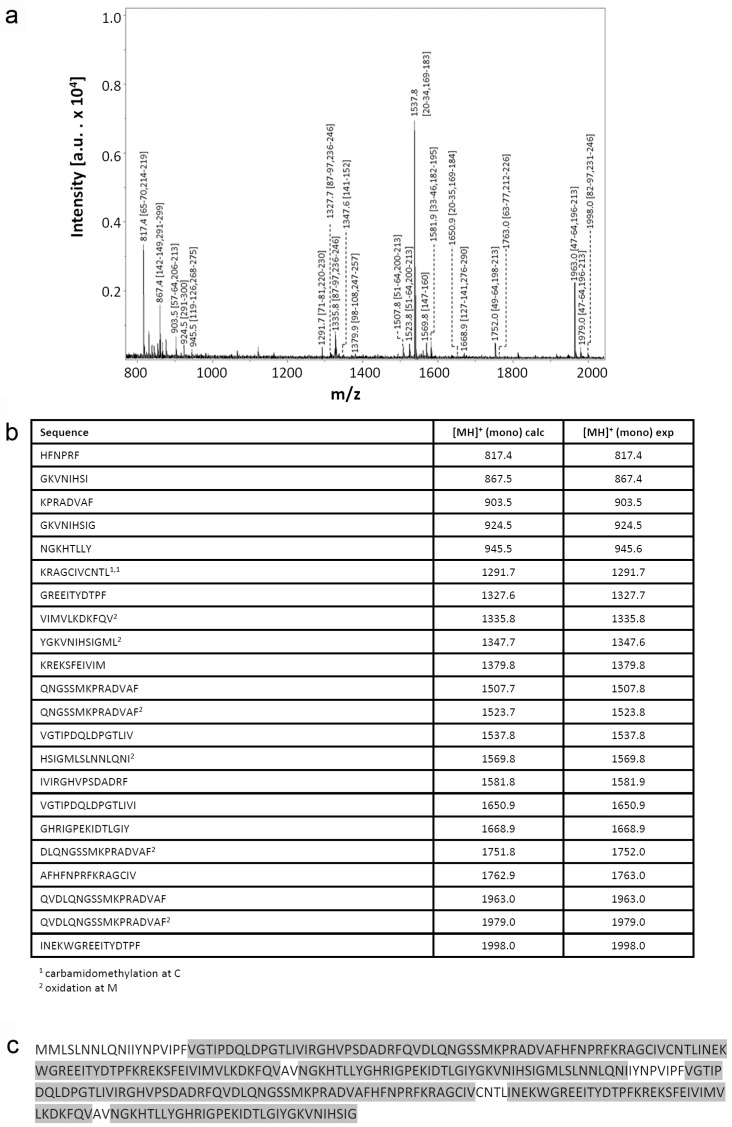
Mass spectrometric fingerprinting of peptides obtained by treatment of Gal-8 (NN) with chymotrypsin. (**a**) The annotated spectrum, (**b**) the list of detected peptides with their calculated (calc) and experimentally (exp) measured mass values and (**c**) the sequence coverage are shown.

Recombinant proteins obtained by extensive re-design require a rigorous analytical processing to verify its assumed biochemical nature. Respective analysis of this variant protein started with determination of its mass. Peaks for singly and doubly charged molecular ions without evidence for any major contamination were recorded (Figure 2a). The measured value of 33,781.9 Da of the singly charged protein is rather close to the calculated mass (33,777.8 Da), as is also the case for wild-type Gal-8S (NC) processed as control (measured: 35,805.4 Da; calculated: 35,807.8 Da) (Figure 2b). Next, mass spectrometric fingerprinting was performed to ascertain the correspondence of protein to cDNA sequence. To obtain a high degree of sequence coverage two rounds of peptide profiling were done after independent treatment of Gal-8 (NN) with trypsin (Figure 3) or with chymotrypsin (Figure 4). The combination of the results of both experiments reached 97% representation of the sequence in resulting peptides. Tryptic digestion of the (wild-type) Gal-8S (NC) protein, performed as control in parallel, covered 92% of the sequence (Supplementary Materials Figure S1). These results extended the evidence for absence of any deviation from the expected structure.

**Figure 5:**
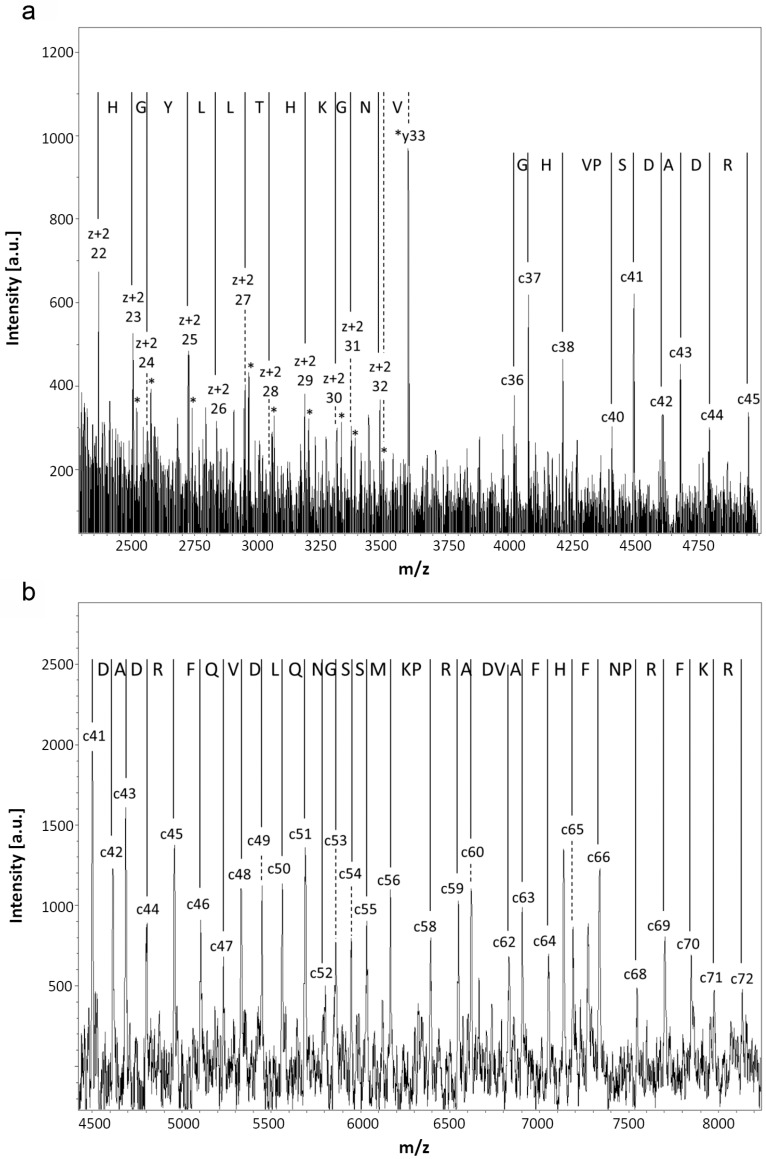
Mass determination and peptide ladder of N- and C-terminal regions by re/linISD for Gal-8 (NN). (**a**) reISD spectrum with the smallest C (left)- and N (right)-terminal peptides and the ensuing peptide ladder; (**b**) linISD spectrum with the smallest detectable N-terminal peptide and the ensuing peptide ladder.

**Figure 6:**
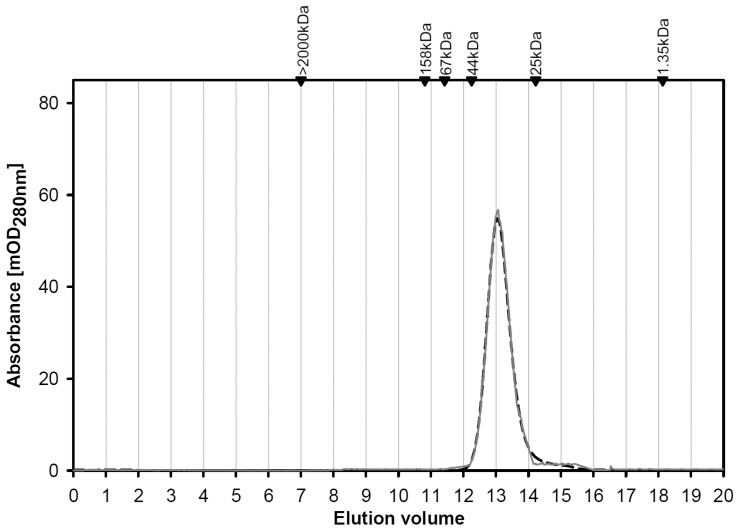
Elution profiles of Gal-8 (NN) during gel filtration in the absence and presence of 40 mM lactose. Black arrowheads mark positions of elution of molecular weight markers.

As an additional means to collect mass information on N- and C-terminal regions by stepwise sequencing, starting from the smallest obtained c- or (z + 2) ions, respectively, spectra of reflectron in-source decay (reISD) (Figure 5a) and linISD (Figure 5b) were recorded. At the C-terminus, the peptide ladder started at (z + 2) 22/23, at the N-terminus at c36/c37 (reISD) and c42/43, reaching the c71/72 positions (lin(ear)ISD). Listing of the calculated and measured masses of the c, z + 2, and y-ions is given in Supplementary Materials Table S1, solidifying the evidence for the assumed error/substitution-free product nature. The presented collective experimental information excluded both any deviation in the sequenced stretch from the cDNA-based template and a post-translational modification except for the iodoacetamide-dependent covalent modification of cysteine and oxidation of methionine residues. Determination of the isoelectric point at 9.1 (calculated: 9.06) corroborated this conclusion (Supplementary Materials Figure S2). In gel filtration, the variant protein gave a single peak at the position predicted for the monomer status irrespective of the presence of lactose (40 mM) (Figure 6). These data documented the purity and quaternary structure and enabled us to proceed with study of the variant’s profile of glycan specificity in an array and in FACScan analysis, as well as its activity as agglutinin and a cell growth regulator.

#### 2.1.3. Glycan Specificity Profile

Following biotinylation under activity-preserving conditions, the variant Gal-8 (NN) was first tested in a solid-phase assay with a glycoprotein without/with *N*-glycan α2,3-sialylations (fetuin/asialofetuin). The ligand was adsorbed to the plastic surface of microtiter plate wells, and assays with biotinylated lectin revealed saturable and carbohydrate-inhibitable binding at K_d_-values of 148 ± 29 nM (asialofetuin) and 93 ± 10 nM (fetuin with α2,3-sialylation in the α1,6-arm and the β1,4-branch of the α1,3-arm [50]). These results, as the resin-based purification did, revealed the ability of the labeled variant to bind to surface-presented glycocompounds so that performing array-based analysis was possible. As shown in Table 1, strong signals were observed mostly for the sulfated trisaccharide Neu5Acα2,3Galβ1,3(6-*O*-Su)-GlcNAc, 3′-sialyllactose, the GD3 tetrasaccharide, and other 3′-sialylated saccharides. These glycans exhibited binding properties for the wild-type protein, too, under these conditions. The exchange of the N/C-CRDs, however, led to a nearly complete abrogation of binding of Gal-8 (NN) to histo-blood group ABH determinants, a typical feature of the C-terminal CRD of Gal-8 (NC).

#### 2.1.4. Cell Binding and Aggregation

**Figure 7:**
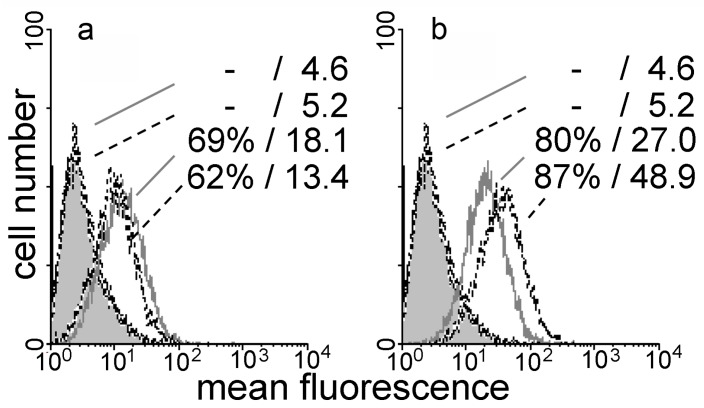
Flow cytofluorometric analysis of binding of labeled Gal-8 (NN) (40 μg/mL) (**a**) and Gal-8 (NC) (20 μg/mL); (**b**) to the surface of parental (grey line) and of neuraminidase-treated CHO cells (dashed line). Numbers in each panel denote percentage of positive cells/mean fluorescence intensity for controls (without/with neuraminidase treatment) and the respective lectin-dependent stainings. Control profiles for lectin-independent background are shown as grey area.

Moving from glycan presentation on an array to cell surfaces (CHO cells with their abundant α2,3-sialylation), respective processing with the labeled variant protein led to a signal (Figure 7a). The variant thus retains binding to cells, a typical feature of wild-type galectins, and changes in the glycan profile can affect binding, e.g., in the status of sialylation [51]. The intensity of cell staining by this variant was reduced by enzymatic desialylation (Figure 7a). This process does not abolish binding but makes terminal *N*-acetyllactosamine (LacNAc) accessible which can still act as a ligand. Their presentation engendered enhanced binding of the wild-type protein, especially seen in mean fluorescence intensity (Figure 7b). Association to the surface of a cell can also make bridging (in *trans*) possible. In this assay type, the label-free protein is tested for its ability to aggregate cells. As the wild-type protein does, the variant acts as an agglutinin. Rabbit erythrocytes were aggregated at the minimal concentration of 0.3 μg/50 μL (1.25 μg/mL for the wild-type protein), with 12.5 mM lactose blocking the lectins’ activity. When testing mixtures of the two CRDs (8N + 8C) to show dependence of activity on bivalency, a concentration of 20 μg/mL was required for agglutination. In the case of human erythrocytes, positivity was observed at 1.25 μg/50 μL for both bivalent proteins. Likely reflecting the difference in signal intensity in FACScan analysis, the minimal concentrations for aggregate formation of the CHO cells were 1.7 μg/50 μL for the variant and 0.78 μg/50 μL for Gal-8S. As a measure of post-binding activity, testing of Gal-8S (WT/F19Y) had delineated a negative effect of the natural single nucleotide polymorphism-based Gal-8S (F19Y) form on proliferation of human colon cancer lines (SW480, HCT116) [47]. This single-site deviation from the common sequence thus led to a growth inhibition, posing the question of an impact after domain shuffling. When assayed under identical conditions at 100 μg/mL, presence of the wild-type and variant proteins had no significant influence on cell growth (not shown). In contrast, Gal-8N reduced the cell number by about 40% under identical conditions, and the significant release of lactate dehydrogenase revealed toxicity exerted by the N-type CRD but not its homodimer.

## 3. Discussion

The emerging role of lectins as readers and interpreters of glycan-encoded determinants with biomedical relevance provides ample incentive to delineate structure–activity relationships in detail. The initial focus of engineering structural aspects has been given to altering the quaternary structure. Examined as a role model, tetrameric concanavalin A had first been turned into dimers by succinylation or acetylation [52], which were later made monovalent by partial photoaffinity labeling [53,54]. A different route, that is selective reduction of disulfide bridges between subunits followed by alkylation of the sulfhydryl groups, also led to monomers. They could favorably be employed in flow cytometry due to the loss of capacity for mediating cell aggregation [55,56]. In these cases, the valency of the lectin was deliberately reduced by chemical modification. The same aim was reached by a single-site mutation, and the resulting variant had become instrumental to separate glycan binding from glycan cross-linking, with biomedical potential for blocking viral infection without activating T cells [57]. The growing realization that tissue lectins pair with a fairly small set of functional counterreceptors gives such efforts a direct physiological scope and impact.

As shown in Figure 1 for galectins, two structural parameters define each family member: (i) overall design of the protein, classified into three groups; and (ii) the contact site for glycans in the CRD. These properties are the main features that should underlie the recognition process, a challenge for devising variants. Each type of design has already invited us to take the first step along this way. In the case of homodimeric Gal-1, covalent bridging of the two CRDs by insertion of linkers between them and the combination of the CRD of Gal-1 with a CRD from Gal-9 have turned the proto-type into a tandem-repeat-type protein [58,59,60,61,62]. The N-terminal tail of chimera-type Gal-3 had been stepwisely shortened or used as a platform to present the Gal-8N CRD [63,64,65,66]. Homodimer formation had been performed for Gal-9 (Gal-9 (NN) and Gal-9 (CC)) and revealed similar eosinophil chemoattractant capacities and activities to induce apoptosis in Jurkat T cells for the wild-type and variant proteins [67,68]. The two CRDs of Gal-9, however, do not present such a marked disparity of glycan specificity for 3′-sialylation as Gal-8N/C do [40]. The exceptionally high affinity in the nM range of Gal-8N for sialylated/sulfated β-galactosides was a reason to embark on the engineering of a Gal-8 variant with two 8N CRDs arranged in tandem. Tandem-repeat-type Gal-4 had been engineered with respect to the length of the linker [69,70,71].

Production of the variant of human Gal-8 as soluble protein was possible for the version without linker. The lectin could be purified by affinity chromatography on lactose-bearing resin. The thorough mass spectrometric analysis of the basic protein excluded presence of any post-translational or chemical modification, for example, formation of an adduct with lactose by glycation, found for Gal-3 at Lys176 recently [72]. In solution, Gal-8 (NN) behaved as a monomer under the conditions of gel filtration, its lactose-inhibitable cross-linking activity revealing that both CRDs are active in the product. This molecular architecture led to a profile of strongly binding glycans with sialylated/sulfated β-galactosides and a comparatively drastic reduction of positivity for ABH histo-blood group epitopes. These glycans preferentially interact with Gal-8’s C-CRD [44]. As a consequence, opposite responses were seen in assays on fluorescent cell binding after sialidase treatment, with a decrease for Gal-8 (NN) and increase for Gal-8 (NC). The marked contribution of 6-*O*-sulfation of the GlcNAc moiety in type I LacNAc in the most active glycan for the variant reflects its special role to enhance affinity and selectivity against galectin-1, when testing sulfated LacNAc derivatives with both galectins [73].

In conclusion, the availability and documented activity in cell binding and as agglutinin open the door to define in detail the impact of combining two CRDs with special preferences while maintaining affinity to the canonical β-galactoside LacNAc. Explicitly, comparative testing in assays on cellular uptake and intracellular sorting [74], contact formation between cells, e.g., myeloma/endothelial cells and the extracellular matrix [75,76], and counterreceptor characterization [38,49] are now possible. Furthermore, such experimental work can also help to answer the question why Gal-8N is toxic, whereas Gal-8 (NN) has no such activity on the tested human colon cancer cells. Considering the versatility of branch-end sialylation/sulfation as a recognition signal, generating new tools for detection and isolation of distinct negatively charged glycans has principal merit [77,78,79]. Toward this aim, this variant can serve as platform for further mutational adaption of the human galectin. Interestingly, such a site-specific process implemented strong affinity for α2,6-sialylated *N*-glycans into a galactoside-specific β-trefoil lectin from the earthworm [80], underscoring the promising perspective of lectin engineering [81], from single-site mutations to altering the modular architecture, as shown here.

## 4. Materials and Methods

### 4.1. cDNA Engineering and Protein Production

Establishing the tandem-repeat arrangement of two Gal-8N CRDs on the level of the cDNA started by separate amplifications of cDNAs for Gal-8N without sequence extension, and the Gal-8N CRD was extended by sequences encoding the Gal-8S linker (33 aa) or the Gal-8L linker (75 aa). Following cDNA amplification, sequences were cloned into a bacterial vector so that artificial generation of restriction sites could be exploited to yield the new homodimeric display. In a final step, site-directed mutagenesis was applied to reconstitute wild-type codons at the position of the artificial restriction sites. In detail, PCR amplification was directed for the first N-CRD by the sense primer 5′-CATATGATGTTGTCCTTAAACAACCTAC-3′ with an internal *Nde*I restriction site and the antisense primer 5′-GGTACCAATTGAGTGAATATTCACTTTG-3′ with an internal *Kpn*I restriction site to generate the Gal-8 (NN). Variants with linkers contained respective extensions (33 aa linker: 5′-AGATCTAAGCTGGGGCGTGC-3′, 75 aa linker: 5′-AGATCTTGACACATAGTTCATAGGTG-3′, both sense primers with an internal *Bgl*II restriction site). For the second N-CRD, the sense primer 5′-GGTACCTTGTCCTTAAACAACCTAC-3′ with an internal *Kpn*I restriction site (Gal-8 (NN); variants with linker: 5′-AGATCTTTGTCCTTAAACAACCTACA-3′ with an internal *Bgl*II restriction site) and the antisense primer 5′-GTCGACTCAACCAATTGAGTGAATATT-3′ with an internal *Sal*I restriction site were used.

The amplification products were then propagated in the pGEM-T easy vector (EcoRV-linearized with single 3′T overhangs; Promega, Mannheim, Germany), digestion was performed at the 5′/3′ end with the respective pair of restriction enzymes (*Nde*I*/Kpn*I*, Kpn*I*/Sal*I*, Nde*I*/Bgl*II*, Bgl*II*/Sal*I), and gel extraction led to vector-released cDNAs with sticky ends. Respective cDNAs were combined making use of the artificial restriction site (Gal-8 (NN): *Kpn*I; linker versions: *Bgl*II) and ligated into a pET-24a plasmid (Novagen, Darmstadt, Germany). In the final step of engineering, the pET-24a plasmids containing the complete cDNAs encoding the homodimeric Gal-8 variants (900 bp for Gal-8 (NN), 999 bp for this protein with the 33 aa-long linker and 1125 bp for the protein version with the 75 aa-long linker) were then template in a modified QuikChange^®^ site-directed mutagenesis procedure (Agilent Technologies, Waldbronn, Germany) to revert codon sequences of the artificial restriction sites to the wild-type codons. Resulting plasmids were isolated from kanamycin-resistant colonies grown on LB agar plates and correct sequences ascertained by commercial DNA sequencing. Recombinant protein production was done in the pET-24a (pGEMEX-1; Promega)/*Escherichia coli* strain BL21(DE3)pLysS/Rosetta^TM^(DE3)pLysS system with TB medium (Roth, Karlsruhe, Germany), systematically testing parameters (after an initial growth phase of 4–5 h at 37 °C up to an OD of 600 nm of 0.6–0.8), i.e., the temperature at 22 °C, 30 °C and 37 °C and the final IPTG concentrations of 75 μM, 100 μM, and 200 μM. Presence of the protein in the soluble fraction was monitored by analysis using gel electrophoresis and Western blotting with a home-made polyclonal anti-Gal-8 antibody preparation after extract separation into soluble and pellet fractions as described [82]. Soluble protein was purified by affinity chromatography on lactose-Sepharose 4B as crucial step, as previously described for human and chicken galectin-8 [47,82].

### 4.2. Analytical Procedures

Matrix-assisted laser desorption/ionization time-of-flight (TOF) mass spectrometry on an Ultraflex TOFTOF I instrument (Bruker Daltonik, Bremen, Germany) equipped with a nitrogen laser (20 Hz) was performed for the intact protein in the positive-ion linear mode with ion acceleration voltage at 25 kV and first extraction plate at 23 kV. Peptide fingerprinting was done in the positive-ion reflectron mode at reflector voltage of 26.3 kV and 21.75 kV at the first extraction plate. Proteolytic cleavage by trypsin and chymotrypsin was carried out in 40 mM NH_4_HCO_3_ or 100 mM Tris-HCl (pH 7.8), respectively, starting with 10 μg of protein dissolved in 10 μL digestion buffer. Following routine treatment for reduction of disulfide bridges by dithiothreitol (DTT) and alkylation of any resulting thiol groups by iodoacetamide, 100 ng trypsin (overnight at 37 °C) or 100 ng chymotrypsin (3 h at 25 °C) were applied, followed by desalting the solution using zip-tip C18 (Merck Millipore, Darmstadt, Germany) according to the manufacturer’s instructions. The peptides were eluted with 2 μL saturated solution of α-cyano-4-hydroxy-cinnamic acid in 50% acetonitrile in 0.1% TFA (TFA50), 1 μL pipetted on the MALDI target followed by 1 μL of the TFA50 solution. The top-down approach of protein characterization by ISD used sinapinic acid as matrix, as described [62,66]. Settings for linISD in the positive-ion linear mode were 25 kV for ion acceleration and 23.2 kV at the first extraction plate, for reISD 21.75 kV at the first extraction plate and a reflector voltage at 26.3 kV. Data acquisition following up to 5000 individual laser shots, calibration procedures including instrument control, and data analysis including processing annotated spectra by BioTools 3.0 (Bruker Daltonik) were done as described [62,65]. The isolelectric point was determined by two-dimensional gel electrophoresis after dissolving 10 μg protein in 155 μL of a solution of 8 M urea, 20 mM DTT and 2% CHAPS, loading the sample on an IEF-strip (Zoom IPG strip, pH 6–10; Thermo Fisher Scientific, Dreieich, Germany), and running the gel in a ZOOM IPG Runner Cell. For the second dimension, a NuPAGE Novex 4–12% Bis-Tris gel (Thermo Fisher Scientific) was applied. Finally, the gel was Coomassie stained. The theoretical pI value was calculated with the “ExPASy Compute pI tool” (ExPASy, http://web.expasy.org/compute_pi/). Gel filtration (100 μg of protein in 50 μL buffer) was performed on a calibrated Superose HR10/30 column using an ÄKTA purifier 10 system (GE Healthcare, Munich, Germany) at 4 °C and a flow rate of 0.5 mL/min.

### 4.3. Glycan Array

Arrays produced by printing glycans (50 μM; total of 416 oligosaccharides) were from Semiotik LLC (Moscow, Russia). Gal-8 (NN/NC), labeled by conjugation of biotin using the *N*-hydroxysuccinimide ester derivative (Sigma, Munich, Germany) under activity-preserving conditions as described [83,84,85], was tested at the concentration of 50 μg/mL in phosphate-buffered saline (PBS) containing 0.1% Tween-20 and 1% bovine serum albumin. This solution was incubated for 1 h at 37 °C in a humidified chamber. The chips had been pretreated with PBS containing 0.1% Tween-20 for 15 min. After thorough washing to remove the labeled protein, probing with streptavidin labeled with the ALEXA Fluor^®^555 dye (Thermo Fisher Scientific) followed for 45 min at 22 °C. Washing with PBS containing 0.001% Tween-20 and then with deionized water removed the fluorescent reagent before chips were scanned on a Innoscan 1100 AL scanner (Innopsys, Carbonne, France) using an excitation wavelength of 543 nm at 10 μm resolution. Data on six spots per test compound on the chip surface were processed using ScanArray Express 4.0 software and the fixed 70 μm-diameter circle method as well as Microsoft Excel, as described [86,87]. The results are reported as median RFU (relative fluorescence units) of replicates. Median deviation was measured as interquartile range. Any signal whose fluorescence intensity exceeded the background value by a factor of five (signals from ligand-free areas were counted as a background) was considered as significant.

### 4.4. Solid-Phase and Cell Assays

Dissociation constants of binding of biotinylated Gal-8 (NN) to the *N*-glycans of glycoproteins (fetuin and the chemically desialylated asialofetuin) were determined in microtiter plate wells presenting surface-adsorbed ligand (after overnight incubation at 4 °C of solution at 0.5 μg/50 μL) and Scatchard analysis, as described [88]. Carbohydrate-dependent galectin binding to the surface of parental Chinese hamster ovary (CHO) cells, kindly provided from P. Stanley (Albert Einstein College of Medicine, Bronx, NY, USA), by flow cytofluorometry using streptavidin/R-phycoerythrin as fluorescent indicator (1:40; Sigma, Munich, Germany) was determined without/with treatment with *C. perfringens* neuraminidase (0.01 U in 50 μL PBS for 2 × 10^5^ cells at 37 °C for 1 h; ROCHE, Mannheim, Germany) as described [51,89]. Haemagglutination of trypsin-treated, glutaraldehyde-fixed rabbit and human erythrocytes was analyzed in 96-well (V-shaped) microtiter plates using 2-fold serial dilutions as determined [90]. Aggregation of CHO cells was analyzed by microscopic assessment. Growth of cells of the human colon adenocarcinoma lines HCT116 and SW480 in Dulbecco’s minimal essential medium containing 10% fetal bovine serum was quantitated in parallel assays using a commercial kit (CellTiter 96, Promega), as described [47].

## Figures and Tables

**Table 1:** Listing of the most active ligands of labeled Gal-8 (NN) and signal intensities.

Structure	Median	MAD ^#^
Neu5Acα2,3Galβ1,3(6-*O*-Su)GlcNAc	59,796	1931
Neu5Acα2,3Galβ1,4Glc-Gly-Phe *	43,636	4717
Neu5Acα2,8Neu5Acα2,3Galβl,4Glc	31,204	1141
Neu5Aα2,3Galβ1,3GlcNAcβ1,3Galβ1,4Glc	18,278	6705
Neu5Acα2,3Galβ1,3GlcNAc	17,215	1619
Neu5Acα2,3Galβ1,4Glc	8888	294
3-*O*-Su-Galβ1,4(6-*O*-Su)Glc	5279	146

* Analogs with other hydrophobic amino acids have similar potency; ^#^ mean absolute deviation.

## Supplementary Materials

The following are available online. Figure S1: Mass spectrometric fingerprinting of peptides obtained by treatment of Gal-8S (NC) with trypsin; Table S1a: Calculated and experimental masses of c-ions observed in the reISD spectra for Gal-8 (NN); Table S1b: Calculated and experimental masses of z + 2-ions observed in the reISD spectra for Gal-8 (NN).

## References

[1] Kopitz J., Gabius H.-J. (2009). Glycolipids. The Sugar Code: Fundamentals of Glycosciences.

[2] Zuber C., Roth J., Gabius H.-J. (2009). *N*-Glycosylation. The Sugar Code: Fundamentals of Glycosciences.

[3] Corfield A.P., Berry M. (2015). Glycan variation and evolution in the eukaryotes. Trends Biochem. Sci..

[4] Kudelka M.R., Ju T., Heimburg-Molinaro J., Cummings R.D. (2015). Simple sugars to complex disease: Mucin-type *O*-glycans in cancer. Adv. Cancer Res..

[5] Schengrund C.-L. (2015). Gangliosides: Glycosphingolipids essential for normal neural development and function. Trends Biochem. Sci..

[6] Corfield A. (2017). Eukaryotic protein glycosylation: A primer for histochemists and cell biologists. Histochem. Cell Biol..

[7] Kopitz J. (2017). Lipid glycosylation: A primer for histochemists and cell biologists. Histochem. Cell Biol..

[8] Nakagawa H., Gabius H.-J. (2009). Analytical aspects: Analysis of protein-bound glycans. The Sugar Code: Fundamentals of Glycosciences.

[9] Nishimura S.-I. (2011). Toward automated glycan analysis. Adv. Carbohydr. Chem. Biochem..

[10] Lazar I.M., Lee W., Lazar A.C. (2013). Glycoproteomics on the rise: Established methods, advanced techniques, sophisticated biological applications. Electrophoresis.

[11] Novotny M.V., Alley W.R., Mann B.F. (2013). Analytical glycobiology at high sensitivity: Current approaches and directions. Glycoconj. J..

[12] Gabius H.-J., Roth J. (2017). An introduction to the sugar code. Histochem. Cell Biol..

[13] Roth J. (1978). The lectins: Molecular probes in cell biology and membrane research. Exp. Pathol..

[14] Caselitz J. (1987). Lectins and blood group substances as “tumor markers”. Curr. Top. Pathol..

[15] Danguy A., Akif F., Pajak B., Gabius H.-J. (1994). Contribution of carbohydrate histochemistry to glycobiology. Histol. Histopathol..

[16] Drake P.M., Cho W., Li B., Prakobphol A., Johansen E., Anderson N.L., Regnier F.E., Gibson B.W., Fisher S.J. (2010). Sweetening the pot: Adding glycosylation to the biomarker discovery equation. Clin. Chem..

[17] André S., Kaltner H., Manning J.C., Murphy P.V., Gabius H.-J. (2015). Lectins: Getting familiar with translators of the sugar code. Molecules.

[18] Solís D., Bovin N.V., Davis A.P., Jiménez-Barbero J., Romero A., Roy R., Smetana K., Gabius H.-J. (2015). A guide into glycosciences: How chemistry, biochemistry and biology cooperate to crack the sugar code. Biochim. Biophys. Acta.

[19] Tang H., Hsueh P., Kletter D., Bern M., Haab B. (2015). The detection and discovery of glycan motifs in biological samples using lectins and antibodies: New methods and opportunities. Adv. Cancer Res..

[20] Manning J.C., Romero A., Habermann F.A., García Caballero G., Kaltner H., Gabius H.-J. (2017). Lectins: A primer for histochemists and cell biologists. Histochem. Cell Biol..

[21] Kilpatrick D.C. (2002). Animal lectins: A historical introduction and overview. Biochim. Biophys. Acta.

[22] Gabius H.-J., Kaltner H., Kopitz J., André S. (2015). The glycobiology of the CD system: A dictionary for translating marker designations into glycan/lectin structure and function. Trends Biochem. Sci..

[23] Gabius H.-J., Manning J.C., Kopitz J., André S., Kaltner H. (2016). Sweet complementarity: The functional pairing of glycans with lectins. Cell. Mol. Life Sci..

[24] Von der Lieth C.-W., Siebert H.-C., Kozár T., Burchert M., Frank M., Gilleron M., Kaltner H., Kayser G., Tajkhorshid E., Bovin N.V. (1998). Lectin ligands: New insights into their conformations and their dynamic behavior and the discovery of conformer selection by lectins. Acta Anat..

[25] Gabius H.-J., André S., Jiménez-Barbero J., Romero A., Solís D. (2011). From lectin structure to functional glycomics: Principles of the sugar code. Trends Biochem. Sci..

[26] Lee Y.C., Lee R.T., Rice K.G., Ichikawa Y., Wong T.C. (1991). Topography of binding sites of animal lectins: Ligands’ view. Pure Appl. Chem..

[27] Chabre Y.M., Roy R., Gabius H.-J. (2009). The chemist’s way to prepare multivalency. The Sugar Code: Fundamentals of Glycosciences.

[28] Murphy P.V., André S., Gabius H.-J. (2013). The third dimension of reading the sugar code by lectins: Design of glycoclusters with cyclic scaffolds as tools with the aim to define correlations between spatial presentation and activity. Molecules.

[29] Cecioni S., Imberty A., Vidal S. (2015). Glycomimetics versus multivalent glycoconjugates for the design of high affinity lectin ligands. Chem. Rev..

[30] Roy R., Murphy P.V., Gabius H.-J. (2016). Multivalent carbohydrate-lectin interactions: How synthetic chemistry enables insights into nanometric recognition. Molecules.

[31] Hirabayashi J. (1997). Recent topics on galectins. Trends Glycosci. Glycotechnol..

[32] Cooper D.N.W. (2002). Galectinomics: Finding themes in complexity. Biochim. Biophys. Acta.

[33] Kaltner H., Toegel S., García Caballero G., Manning J.C., Ledeen R.W., Gabius H.-J. (2017). Galectins: Their network and roles in immunity/tumor growth control. Histochem. Cell Biol..

[34] Zick Y., Eisenstein M., Goren R.A., Hadari Y.R., Levy Y., Ronen D. (2004). Role of galectin-8 as a modulator of cell adhesion and cell growth. Glycoconj. J..

[35] Cludts S., Decaestecker C., Mahillon V., Chevalier D., Kaltner H., André S., Remmelink M., Leroy X., Gabius H.-J., Saussez S. (2009). Galectin-8 up-regulation during hypopharyngeal and laryngeal tumor progression and comparison with galectins-1, -3 and -7. Anticancer Res..

[36] Cattaneo V., Tribulatti M.V., Campetella O. (2011). Galectin-8 tandem-repeat structure is essential for T-cell proliferation but not for co-stimulation. Biochem. J..

[37] Cattaneo V., Tribulatti M.V., Carabelli J., Carestia A., Schattner M., Campetella O. (2014). Galectin-8 elicits pro-inflammatory activities in the endothelium. Glycobiology.

[38] Vinik Y., Shatz-Azoulay H., Vivanti A., Hever N., Levy Y., Karmona R., Brumfeld V., Baraghithy S., Attar-Lamdar M., Boura-Halfon S. (2015). The mammalian lectin galectin-8 induces RANKL expression, osteoclastogenesis, and bone mass reduction in mice. eLife.

[39] Houzelstein D., Gonçalves I.R., Fadden A.J., Sidhu S.S., Cooper D.N.W., Drickamer K., Leffler H., Poirier F. (2004). Phylogenetic analysis of the vertebrate galectin family. Mol. Biol. Evol..

[40] Hirabayashi J., Hashidate T., Arata Y., Nishi N., Nakamura T., Hirashima M., Urashima T., Oka T., Futai M., Müller W.E.G. (2002). Oligosaccharide specificity of galectins: A search by frontal affinity chromatography. Biochim. Biophys. Acta.

[41] Ideo H., Seko A., Ishizuka I., Yamashita K. (2003). The N-terminal carbohydrate recognition domain of galectin-8 recognizes specific glycosphingolipids with high affinity. Glycobiology.

[42] Ideo H., Matsuzaka T., Nonaka T., Seko A., Yamashita K. (2011). Galectin-8-N-domain recognition mechanism for sialylated and sulfated glycans. J. Biol. Chem..

[43] Carlsson S., Oberg C.T., Carlsson M.C., Sundin A., Nilsson U.J., Smith D., Cummings R.D., Almkvist J., Karlsson A., Leffler H. (2007). Affinity of galectin-8 and its carbohydrate recognition domains for ligands in solution and at the cell surface. Glycobiology.

[44] Stowell S.R., Arthur C.M., Slanina K.A., Horton J.R., Smith D.F., Cummings R.D. (2008). Dimeric Galectin-8 induces phosphatidylserine exposure in leukocytes through polylactosamine recognition by the C-terminal domain. J. Biol. Chem..

[45] Vokhmyanina O.A., Rapoport E.M., André S., Severov V.V., Ryzhov I., Pazynina G.V., Korchagina E., Gabius H.-J., Bovin N.V. (2012). Comparative study of the glycan specificities of cell-bound human tandem-repeat-type galectins-4, -8 and -9. Glycobiology.

[46] Yoshida H., Yamashita S., Teraoka M., Itoh A., Nakakita S., Nishi N., Kamitori S. (2012). X-ray structure of a protease-resistant mutant form of human galectin-8 with two carbohydrate recognition domains. FEBS J..

[47] Ruiz F.M., Scholz B.A., Buzamet E., Kopitz J., André S., Menéndez M., Romero A., Solís D., Gabius H.-J. (2014). Natural single amino acid polymorphism (F19Y) in human galectin-8: Detection of structural alterations and increased growth-regulatory activity on tumor cells. FEBS J..

[48] Ruiz F.M., Gilles U., Lindner I., André S., Romero A., Reusch D., Gabius H.-J. (2015). Combining crystallography and hydrogen-deuterium exchange to study galectin-ligand complexes. Chem. Eur. J..

[49] Romaniuk M.A., Tribulatti M.V., Cattaneo V., Lapponi M.J., Molinas F.C., Campetella O., Schattner M. (2010). Human platelets express and are activated by galectin-8. Biochem. J..

[50] Joziasse D.H., Schiphorst W.E.C.M., van den Eijnden D.H., van Kuik J.A., van Halbeek H., Vliegenthart J.F.G. (1987). Branch specificity of bovine colostrum CMP-sialic acid: Galβ1→4GlcNAc-R α2→6-sialyltransferase. Sialylation of bi-, tri-, and tetraantennary oligosaccharides and glycopeptides of the *N*-acetyllactosamine type. J. Biol. Chem..

[51] Amano M., Eriksson H., Manning J.C., Detjen K.M., André S., Nishimura S.-I., Lehtiö J., Gabius H.-J. (2012). Tumour suppressor p16^INK4a^: Anoikis-favouring decrease in N/O-glycan/cell surface sialylation by down-regulation of enzymes in sialic acid biosynthesis in tandem in a pancreatic carcinoma model. FEBS J..

[52] Gunther G.R., Wang J.L., Yahara I., Cunningham B.A., Edelman G.M. (1973). Concanavalin A derivatives with altered biological activities. Proc. Natl. Acad. Sci. USA.

[53] Fraser A.R., Hemperly J.J., Wang J.L., Edelman G.M. (1976). Monovalent derivatives of concanavalin A. Proc. Natl. Acad. Sci. USA.

[54] Beppu M., Terao T., Osawa T. (1979). Covalently cross-linked monovalent, divalent, and tetravalent derivatives of concanavalin A. J. Biochem..

[55] Kurokawa T., Tsuda M., Sugino Y. (1976). Purification and characterization of a lectin from *Wistaria floribunda* seeds. J. Biol. Chem..

[56] Kaku H., Shibuya N. (1992). Preparation of a stable subunit of Japanese elderberry (*Sambucus sieboldiana*) bark lectin and its application for the study of cell surface carbohydrates by flow cytometry. FEBS Lett..

[57] Swanson M.D., Boudreaux D.M., Salmon L., Chugh J., Winter H.C., Meagher J.L., André S., Murphy P.V., Oscarson S., Roy R. (2015). Engineering a therapeutic lectin by uncoupling mitogenicity from antiviral activity. Cell.

[58] Bättig P., Saudan P., Gunde T., Bachmann M.F. (2004). Enhanced apoptotic activity of a structurally optimized form of galectin-1. Mol. Immunol..

[59] Bi S., Earl L.A., Jacobs L., Baum L.G. (2008). Structural features of galectin-9 and galectin-1 that determine distinct T cell death pathways. J. Biol. Chem..

[60] Earl L.A., Bi S., Baum L.G. (2011). Galectin multimerization and lattice formation are regulated by linker region structure. Glycobiology.

[61] Zhang S., Moussodia R.-O., Murzeau C., Sun H.J., Klein M.L., Vértesy S., André S., Roy R., Gabius H.-J., Percec V. (2015). Dissecting molecular aspects of cell interactions using glycodendrimersomes with programmable glycan presentation and engineered human lectins. Angew. Chem. Int. Ed..

[62] Vértesy S., Michalak M., Miller M.C., Schnölzer M., André S., Kopitz J., Mayo K.H., Gabius H.-J. (2015). Structural significance of galectin design: Impairment of homodimer stability by linker insertion and partial reversion by ligand presence. Protein Eng. Des. Sel..

[63] Gong H.C., Honjo Y., Nangia-Makker P., Hogan V., Mazurak N., Bresalier R.S., Raz A. (1999). The NH_2_ terminus of galectin-3 governs cellular compartmentalization and functions in cancer cells. Cancer Res..

[64] Menon R.P., Hughes R.C. (1999). Determinants in the N-terminal domains of galectin-3 for secretion by a novel pathway circumventing the endoplasmic reticulum-Golgi complex. Eur. J. Biochem..

[65] Kopitz J., Vértesy S., André S., Fiedler S., Schnölzer M., Gabius H.-J. (2014). Human chimera-type galectin-3: Defining the critical tail length for high-affinity glycoprotein/cell surface binding and functional competition with galectin-1 in neuroblastoma cell growth regulation. Biochimie.

[66] Ludwig A.K., Vértesy S., Michalak M., Manning J.C., André S., Kübler D., Kopitz J., Kaltner H., Gabius H.-J. (2016). Playing modular puzzle with adhesion/growth-regulatory galectins: Design and testing of a hybrid to unravel structure-activity relationships. Protein Pept. Lett..

[67] Sato M., Nishi N., Shoji H., Seki M., Hashidate T., Hirabayashi J., Kasai K.-I., Hata Y., Suzuki S., Hirashima M. (2002). Functional analysis of the carbohydrate recognition domains and a linker peptide of galectin-9 as to eosinophil chemoattractant activity. Glycobiology.

[68] Lu L.H., Nakagawa R., Kashio Y., Ito A., Shoji H., Nishi N., Hirashima M., Yamauchi A., Nakamura T. (2007). Characterization of galectin-9-induced death of Jurkat T cells. J. Biochem..

[69] Göhler A., André S., Kaltner H., Sauer M., Gabius H.-J., Doose S. (2010). Hydrodynamic properties of human adhesion/growth-regulatory galectins studied by fluorescence correlation spectroscopy. Biophys. J..

[70] Kopitz J., Ballikaya S., André S., Gabius H.-J. (2012). Ganglioside GM1/galectin-dependent growth regulation in human neuroblastoma cells: Special properties of bivalent galectin-4 and significance of linker length for ligand selection. Neurochem. Res..

[71] André S., Wang G.N., Gabius H.-J., Murphy P.V. (2014). Combining glycocluster synthesis with protein engineering: An approach to probe into the significance of linker length in a tandem-repeat-type lectin (galectin-4). Carbohydr. Res..

[72] Jovanović M., Peter-Katalinić J. (2017). Preliminary mass spectrometry characterization studies of galectin-3 samples, prior to carbohydrate-binding studies using affinity mass spectrometry. Rapid Commun. Mass Spectrom..

[73] Tu Z., Hsieh H.W., Tsai C.M., Hsu C.W., Wang S.G., Wu K.J., Lin K.I., Lin C.H. (2013). Synthesis and characterization of sulfated Gal-β1,3/4-GlcNAc disaccharides through consecutive protection/glycosylation steps. Chem. Asian J..

[74] Carlsson S., Carlsson M.C., Leffler H. (2007). Intracellular sorting of galectin-8 based on carbohydrate fine specificity. Glycobiology.

[75] Levy Y., Arbel-Goren R., Hadari Y.R., Eshhar S., Ronen D., Elhanany E., Geiger B., Zick Y. (2001). Galectin-8 functions as a matricellular modulator of cell adhesion. J. Biol. Chem..

[76] Friedel M., André S., Goldschmidt H., Gabius H.-J., Schwartz-Albiez R. (2016). Galectin-8 enhances adhesion of multiple myeloma cells to vascular endothelium and is an adverse prognostic factor. Glycobiology.

[77] Hu D., Huang H., Tateno H., Nakakita S., Sato T., Narimatsu H., Yao X., Hirabayashi J. (2015). Engineering of a 3’-sulpho-Galβ1-4GlcNAc-specific probe by a single amino acid substitution of a fungal galectin. J. Biochem..

[78] Bhide G.P., Colley K.J. (2017). Sialylation of *N*-glycans: Mechanism, cellular compartmentalization and function. Histochem. Cell Biol..

[79] Roy R., Cao Y., Kaltner H., Kottari N., Shiao T.C., Belkhadem K., André S., Manning J.C., Murphy P.V., Gabius H.-J. (2017). Teaming up synthetic chemistry and histochemistry for activity screening in galectin-directed inhibitor design. Histochem. Cell Biol..

[80] Yabe R., Itakura Y., Nakamura-Tsuruta S., Iwaki J., Kuno A., Hirabayashi J. (2009). Engineering a versatile tandem repeat-type α2-6sialic acid-binding lectin. Biochem. Biophys. Res. Commun..

[81] Hu D., Tateno H., Hirabayashi J. (2015). Lectin engineering, a molecular evolutionary approach to expanding the lectin utilities. Molecules.

[82] Kaltner H., Solís D., André S., Lensch M., Manning J.C., Mürnseer M., Sáiz J.L., Gabius H.-J. (2009). Unique chicken tandem-repeat-type galectin: Implications of alternative splicing and a distinct expression profile compared to those of the three proto-type proteins. Biochemistry.

[83] Gabius H.-J., Wosgien B., Hendrys M., Bardosi A. (1991). Lectin localization in human nerve by biochemically defined lectin-binding glycoproteins, neoglycoprotein and lectin-specific antibody. Histochemistry.

[84] Kaltner H., García Caballero G., Sinowatz F., Schmidt S., Manning J.C., André S., Gabius H.-J. (2016). Galectin-related protein: An integral member of the network of chicken galectins. 2. From expression profiling to its immunocyto- and histochemical localization and application as tool for ligand detection. Biochim. Biophys. Acta.

[85] Manning J.C., García Caballero G., Knospe C., Kaltner H., Gabius H.-J. (2017). Network analysis of adhesion/growth-regulatory galectins and their binding sites in adult chicken retina and choroid. J. Anat..

[86] García Caballero G., Flores-Ibarra A., Michalak M., Khasbiullina N., Bovin N.V., André S., Manning J.C., Vértesy S., Ruiz F.M., Kaltner H. (2016). Galectin-related protein: An integral member of the network of chicken galectins. 1. From strong sequence conservation of the gene confined to vertebrates to biochemical characteristics of the chicken protein and its crystal structure. Biochim. Biophys. Acta.

[87] García Caballero G., Kaltner H., Michalak M., Shilova N., Yegres M., André S., Ludwig A.K., Manning J.C., Schmidt S., Schnölzer M. (2016). Chicken GRIFIN: A homodimeric member of the galectin network with canonical properties and a unique expression profile. Biochimie.

[88] André S., Kaltner H., Lensch M., Russwurm R., Siebert H.-C., Fallsehr C., Tajkhorshid E., Heck A.J.R., von Knebel-Döberitz M., Gabius H.-J. (2005). Determination of structural and functional overlap/divergence of five proto-type galectins by analysis of the growth-regulatory interaction with ganglioside GM1 in silico and in vitro on human neuroblastoma cells. Int. J. Cancer.

[89] André S., Sansone F., Kaltner H., Casnati A., Kopitz J., Gabius H.-J., Ungaro R. (2008). Calix[*n*]arene-based glycoclusters: Bioactivity of thiourea-linked galactose/lactose moieties as inhibitors of binding of medically relevant lectins to a glycoprotein and cell-surface glycoconjugates and selectivity among human adhesion/growth-regulatory galectins. ChemBioChem.

[90] Gabius H.-J., Engelhardt R., Rehm S., Cramer F. (1984). Biochemical characterization of endogenous carbohydrate-binding proteins from spontaneous murine rhabdomyosarcoma, mammary adenocarcinoma, and ovarian teratoma. J. Natl. Cancer Inst..

