# Supplementary material for: Studying the Structural Significance of Galectin Design by Playing a Modular Puzzle: Homodimer Generation from Human Tandem-Repeat-Type (Heterodimeric) Galectin-8 by Domain Shuffling
Source: Molecules. 2017 Sep 19;22(9):1572. doi: 10.3390/molecules22091572 (PMC6151538; doi:10.3390/molecules22091572)
Supplement: Supplementary file 1 [file molecules-22-01572-s001.pdf]

Figure S1

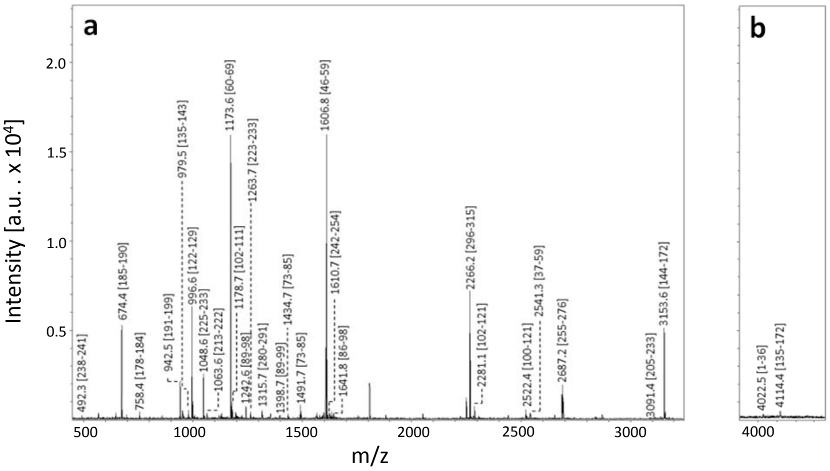

c

**Sequence**	**$[MH]^+$ (mono) calc**	**$[MH]^+$ (mono) exp**
AFVR	492.3	492.3
LPFAAR	674.4	674.4
SGTPQLR	758.4	758.4
LNTPMGPGR	942.5	942.5
IDTLGIYGK	979.5	979.5
HTLLYGHR	996.5	996.6
DIALHLNPR	1048.6	1048.6
SFNVDLLAGK	1063.6	1063.6
ADVAFHFNPR	1173.6	1173.6
SFEIVIMVLK	1178.7	1178.7
EEITYDTPFK	1242.6	1242.6
SKDIALHLNPR	1263.7	1263.7
VAVNGVHSLEYK	1315.7	1315.7
EEITYDTPFKR	1398.7	1398.7
AGCIVCNTLINEK[1]	1434.7	1434.7
AGCIVCNTLINEK[1,1]	1491.7	1491.7
FQVDLQNGSSMKPR	1606.8	1606.8
NSFLQESWGEEER	1610.7	1610.7
WGREEITYDTPFK	1641.8	1641.8
ELSSIDTLEINGDIHLLEVR	2266.2	2266.2
SFEIVIMVLKDKFQVAVNGK[2]	2281.3	2281.1
EKSFEIVIMVLKDKFQVAVNGK	2522.4	2522.4
GHVPSDADRFQVDLQNGSSMKPR	2541.2	2541.3
NITSFPFSPGMYFEMIIYCDVR	2687.2	2687.2
GEVNANAKSFNVDLLAGKSKDIALHLNPR[1]	3091.7	3091.4
VNIHSIGFSFSSDLQSTQASSLELTEISR	3153.6	3153.6
MMLSLNNLQNIIYNPVIPFVGTIPDQLDPGTLIVIR	4022.2	4022.5
IDTLGIYGKVNIHSIGFSFSSDLQSTQASSLELTEISR	4114.1	4114.4

[1] carbamidomethylation at C
[2] oxidation at M

d

MMLSLNNLQNIIYNPVIPFVGTIPDQLDPGTLIVIRGHVPSDADRFQVDLQNGSSMKPRADVAFHFNPRFKRAGCIVCNTLINEK
WGREEITYDTPFKREKSFEIVIMVLKDKFQVAVNGKHTLLYGHRIGPEKIDTLGIYGKVNIHSIGFSFSSDLQSTQASSLELTEISREN
VPKSGTPQLRLPFAARLNTPMGPGRTVVVKGEVNANAKSFNVDLLAGKSKDIALHLNPRLNIKAFVRNSFLQESWGEEERNITS
FPFSPGMYFEMIIYCDVREFKVAVNGVHSLEYKHRFKELSSIDTLEINGDIHLLEVRSW

Supporting Information Figure S1. Mass spectrometric fingerprinting of peptides obtained by treatment of Gal-8S (NC) with trypsin. The annotated spectrum (**a,b**), the list of detected peptides with their calculated (calc) and experimentally (exp) measured mass values (**c**) and the sequence coverage (**d**) are shown.

Figure S2

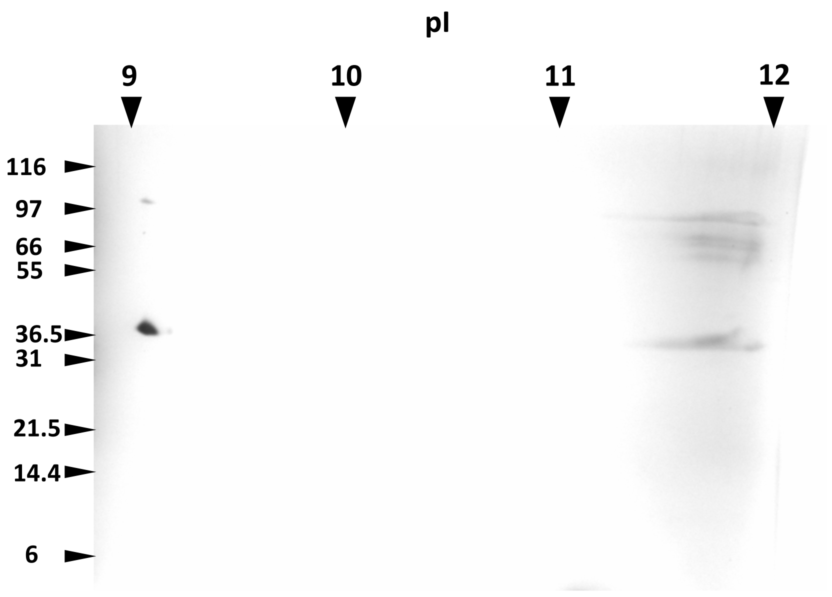

Supporting Information Figure S2. 2D Gel electrophoretic analysis of Gal-8 (NN): protein (10 µg) was dissolved in 155 µl of a solution containing 8 M urea, 20 mM DTT and 2% CHAPS, then loaded on an IEF strip (Zoom IPTG strip pH 9-12; Thermo Fischer Scientific, Dreieich, Germany) and focused in a ZOOM IPG Runner Cell. In the second dimension, a NuPAGE Novex 4-12% Bis-Tris gel (Thermo Fischer Scientific) was applied with a set of marker proteins, as shown on the left side. Finally, the gel was silver stained to reveal the position of the protein.

Table S1a

Calculated and experimental masses of c-ions observed in the reISD spectra for Gal-8 (NN).

c-ions	$[MH]^+$ (av) calc	$[MH]^+$ (av) exp
c36	4023.8	4024.4
c37	4080.8	4080.3
c38	4218.0	4218.7
c39	4317.1	-*
c40	4414.2	4414.3
c41	4501.3	4501.0
c42	4616.4	4616.8
c43	4687.5	4687.1
c44	4802.6	4802.4
c45	4958.7	4958.1

Calculated and experimental masses of c-ions observed in the linISD spectra for Gal-8 (NN).

c-ions	$[MH]^+$ (av) calc	$[MH]^+$ (av) exp
c41	4501.3	4499.8
c42	4616.4	4615.0
c43	4687.5	4685.9
c44	4802.6	4800.7
c45	4958.7	4958.5
c46	5105.9	5105.4
c47	5234.0	5234.0
c48	5333.2	5332.8
c49	5448.3	5448.6
c50	5561.4	5561.9
c51	5689.6	5690.7
c52	5803.7	5802.2
c53	5860.7	5861.6
c54	5947.8	5947.8
c55	6034.9	6034.1
c56	6166.1	6166.4
c57	6294.2	-*
c58	6391.4	6391.4
c59	6547.5	6547.0
c60	6618.6	6618.4
c61	6733.7	-*
c62	6832.8	6831.4
c63	6903.9	6904.4
c64	7051.1	7051.1
c65	7188.2	7187.9
c66	7335.4	7336.2
c67	7449.5	-*
c68	7546.6	7545.9
c69	7702.8	7702.3
c70	7850.0	7849.3
c71	7978.2	7978.0
c72	8134.3	8134.1

* C-ions not observed

Table S1b

Calculated and experimental masses of z+2-ions observed in the reISD spectra for Gal-8 (NN).

z+2-ions	$[MH]^+$ (av) calc	$[MH]^+$ (av) exp
z+2 22	2366.7	2366.5
z+2 23	2503.9	2503.5
z+2 24	2560.9	2560.8
z+2 25	2724.1	2723.7
z+2 26	2837.3	2837.6
z+2 27	2950.4	2950.1
z+2 28	3051.5	3051.6
z+2 29	3188.7	3188.8
z+2 30	3316.8	3316.6
z+2 31	3373.9	3374.2
z+2 32	3488.0	3487.6

Calculated and experimental masses of y-ions observed in the reISD spectra for Gal-8 (NN).

y-ions	$[MH]^+$ (av) calc	$[MH]^+$ (av) exp
y23	2518.9	2518.8
y24	2575.9	2575.8
y25	2739.1	2738.8
y26	2852.3	-*
y27	2965.4	2965.8
y28	3066.5	3066.4
y29	3203.7	3203.8
y30	3331.8	3331.1
y31	3388.9	3388.6
y32	3503.0	3502.3
y33	3602.1	3602.3

* y-ions not observed
